# Wireless localization with diffusion maps

**DOI:** 10.1038/s41598-020-77695-7

**Published:** 2020-11-26

**Authors:** Amin Ghafourian, Orestis Georgiou, Edmund Barter, Thilo Gross

**Affiliations:** 1 University of California Davis, Davis, USA; 2 University of Cyprus, Nicosia, Cyprus; 3 University of Bristol, Bristol, UK; 4 HIFMB, Helmholtz Institute for Functional Marine Biodiversity, Oldenburg, Germany; 5 Carl-Von-Ossietzky University, Oldenburg, Germany; 6 Alfred-Wegener Institute, Helmholtz Centre for Marine and Polar Research, Bremerhaven, Germany

**Keywords:** Electrical and electronic engineering, Applied mathematics

## Abstract

In the Wireless Localization Matching Problem (WLMP) the challenge is to match pieces of equipment with a set of candidate locations based on wireless signal measurements taken by the pieces of equipment. This challenge is complicated by the noise that is inherent in wireless signal measurements. Here we propose the use of diffusion maps, a manifold learning technique, to obtain an embedding of positions and equipment coordinates in a space that enables coordinate comparison and reliable evaluation of assignment quality at very low computational cost. We show that the mapping is robust to noise and using diffusion maps allows for accurate matching in a realistic setting. This suggests that the diffusion-map-based approach could significantly increase the accuracy of wireless localization in applications.

## Introduction

The Internet of Things (IoT) has emerged with the mission of enabling systems of communicating and interacting components with the capability of remote monitoring and control. These systems are designed to smoothly integrate into their surroundings within the context of many applications in urban and environmental monitoring and control, healthcare, industry, and military. The bold vision of the Smart City is built around such IoT systems with billions or even trillions of interconnected devices and sensors^1,2^. The positive impact of such systems on various domains like infrastructure services, environment, and public safety have been demonstrated in numerous case studies^3^.

A particular class of interconnected systems are wireless sensor networks (WSN), i.e. networks of wireless sensor nodes that measure particular physical features of the environment or equipments^4,5^. Each node consists of several components. These are typically microcontroller, a wireless RF transceiver, battery, memory, and sensors to measure quantities such as temperature, luminosity, and vibration.

In order to accurately analyze and use the data received from the sensor nodes in applications it is often important to be able to identify the location of the sensors^4^. While GPS modules can be used for this purpose, this incurs additional production costs and results in extra power consumption of the sensors (about 90 mW). Furthermore, these modules do not perform sufficiently accurately indoors and underground, where there is poor satellite reception^4^.

The limitations of using GPS modules define a need for alternative localization methods. RF-based methods generally fall into the categories of database, range-based, range-free, and angle-based methods^6–16^. In database methods, the most likely location of target devices are inferred through comparing signal features such as Received Signal Strength Indicator (RSSI) with a previously obtained database^8^. Range-based methods are techniques where an estimate of the distance of the target device from a number of known devices, or anchors, through signal strength measurements and then multilateration leads to an estimate of the location of the device^9–11^. Range-free methods utilize the network topology and RF hop count statistics to estimate the location of target devices^14,15^. In angle-based methods, the angle of arrival (AoA) of the signal from a number of antennas are obtained and the position of the device is estimated through triangulation^16^.

These methods are affected by errors resulting from background noise, pathloss, shadowing, wireless multipath fading, non line-of-sight (NLoS), etc. As a result, the methods are often retuned using empirical models^4^. Model parameters need to be adjusted for specific conditions and therefore such retuning can incur additional costs. Some of these methods might also require additional hardware to measure time difference or angle of arrival, for instance. Considering RSSI is natively supported by many transceiver chipsets, it would be desirable to develop a strategy to use RSSI for accurate sensor localization. Ideally, a developed technique should also incur only small computational costs and have a simple implementation, which will make it scalable and easy to adapt in various applications.

An interesting idea was put forward by Keller and Gur^17^ who propose to use a spectral embedding method for localization. This method provides a very elegant approach to the construction of a spatial map from pairwise measurements between nodes. However due to inherent difficulty of the challenge the locations of several nodes need to be known to achieve good results.

A particular incarnation of the wireless localization problem is the so-called Wireless Localization Matching Problem (WLMP)^18^ where the challenge is to match a set of sensor nodes with an a priori known set of sensor locations, called positions. This type of problem arises, for instance, in facilities where a large number of devices need to be installed in specific locations (e.g. smart lights, smoke detectors, thermostats, etc.). In this setting the set of positions is known and documented in the facility blueprint, whereas keeping track of the position at which a particular device is installed (likely by subcontractors) creates a significant management overhead^4,7^. A similar challenge arises in novel approaches to disaster response, where sensor nodes are airdropped, leading to a de facto random distribution of nodes^6,19^. The set of positions of nodes, but not individual node IDs, can then be obtained by aerial imaging such that locating each device again poses a WLMP^18^.

In this paper, we formulate a solution strategy for WLMP, which is in essence a bipartite matching problem between a number of stationary positions and sensor nodes. In our proposed method, we use position coordinates along with RSSI between pairs of nodes to obtain the best matching between nodes and position candidates. The key innovation that makes this matching efficient is the use of diffusion maps to embed the nodal positions in a new Euclidean space where matching can be performed and with the mapping itself allowing for considerable denoising. In contrast to Keller and Gur^17^ the application of the diffusion map to the conceptually simpler WLMP challenge allows us to capitalize strongly on the power of the diffusion map and achieve very accurate results. The proposed solution is versatile and computationally efficient, which makes it an attractive approach for a spectrum of applications.

## The wireless localization matching problem

In WLMP, we consider a network of *M* wireless nodes labeled $n_1, n_2, \ldots , n_M$, and *M* candidate positions $p_1, p_2, \ldots , p_M$. The positions are known, but it is unknown at which position each node is located. Nodes are equipped with radio transceivers and can exchange messages among them and thus RSSI between pairs of nodes can be obtained and sent to a backhaul server for post-processing. The pairwise RSSIs are recorded in matrix $\mathbf{R}$ where $R_{ij}$ is the signal strength between nodes $n_i$ and $n_j$. For localization purposes this can be converted into entry $D_{ij}$ of the distance matrix using a suitable propagation model. Such models often depend on the specific network type and potentially noisy environment under consideration. The server must then match each node $n_i$ to its corresponding position $p_j$ using the RSSI measurements, the propagation model, and the position coordinates. Anchor nodes, i.e. nodes whose positions are known, might be included. In that case, the server needs to match the remaining nodes and positions, possibly taking advantage of the anchor nodes.

## Methods

Matching problems are well studied and powerful algorithms that solve such problems exist. However, before these algorithms can be applied we must formulate a method to quantify how well a set of RSSI measurements match a specific position. A complicating factor is that measurements and positions use different coordinate systems. The RSSI measurements encode sets of pairwise distances, whereas the positions are given directly in terms of physical coordinates, e.g. longitude and latitude. The positions thus live in a low-dimensional (typically 2D) physical space whereas the RSSI measurements live in a high-dimensional data space. This means that the distance estimates from RSSI, the positions, or both need to be mapped onto a different coordinate system before matching algorithms can be applied. The question then arises what coordinate system is most suited to facilitate the subsequent matching.

The key insight used in this paper is that an advantageous coordinate system for the matching problem can be constructed using a method known as the diffusion map^20–24^. The diffusion map is a manifold learning technique that can discover low-dimensional manifolds in datasets. Node positions in space can be thought of as one such dataset in which a one, two, or three-dimensional manifold exists. This is due to the expectation that for the purpose of the localization, node positions can adequately be described in a Euclidean space with as many dimensions. The diffusion map can then be used to find a natural parameterization of this manifold implied by the distances. The matching is then carried out in the new coordinates determined by the diffusion map (explained below). Importantly, this approach only requires information that would typically be available in applications, i.e. locations of candidate positions and signal strength measurements between pairs of nodes.

Given $\mathbf{R}$, we calculate the distance matrix $\mathbf{D}$ using a suitable wireless propagation path loss model. As an example, we adopt here a non-singular version of the Friis transmission equation indicative of the inverse square law

1 $$\begin{aligned} R_{ij}= \frac{1}{D_{ij}^2+0.1}. \end{aligned}$$

We also consider the log-distance path loss model^25^

2 $$\begin{aligned} 10\log _{10}{R_{ij}}= a-10\eta \log _{10}{D_{ij}} \end{aligned}$$

where *a* comes from device specifications and accounts for transmit power, antenna gains, frequency of transmission, and reference path loss. $\eta$ is the path loss exponent that represents effects from the environment clutter and is typically a value between 2 and 4. We will use these two models in the subsequent simulations. In practice, other propagation models could also be adopted such as those specified by the 3rd Generation Partnership Project, or more recent 5G measurements^26^.

We now construct a similarity matrix $\mathbf{C}$ as

3 $$\begin{aligned} C_{ij}= {\left\{ \begin{array}{ll} k(D_{ij}) &{} i \ne j \\ 0 &{} i = j \end{array}\right. } \end{aligned}$$

where *k* is an appropriate kernel. Here we use the Gaussian kernel

4 $$\begin{aligned} k(d)= \exp {\left( -\frac{d^2}{\sigma ^2}\right) } \end{aligned}$$

with

5 $$\begin{aligned} \sigma ^2= \frac{1}{M^2}\sum _{i,j}{D_{ij}}^2 \end{aligned}$$

It is important that a short ranged kernel is used as the most salient information will be contained in the estimated distances between close nodes.

We now regard the similarity matrix as the adjacency matrix of a weighted network and construct the network’s row-normalized Laplacian

6 $$\begin{aligned} L_{ij}= {\left\{ \begin{array}{ll} -\frac{C_{ij}}{\sum _{k}C_{ik}} &{} i \ne j \\ 1 &{} i = j \end{array}\right. } \end{aligned}$$

This matrix is related to the transition matrix of a random walk in a network, and describes diffusion processes on the network nodes^21^.

To construct a natural coordinate system for the nodes we consider spectral properties of $\mathbf{L}$. It is guaranteed that $\mathbf{L}$ has real non-negative eigenvalues. The number of zero eigenvalues is identical to the number of components in the network^27^ and hence we expect to find exactly one zero eigenvalue. The eigenvector corresponding to this eigenvalue does not carry any information and is ignored in the following.

The most relevant eigenvalues for our purpose are the smallest non-zero eigenvalues as they carry information about the major dimensions of the system. We define $i\hbox {th}$ eigenvector to be the eigenvector with the $i\hbox {th}$ smallest non-zero eigenvalue.

We interpret the entries of eigenvectors of $\mathbf{L}$ as coordinates along new coordinate axes^20^. Note that the eigenvectors of the $M\times M$ matrix $\mathbf{L}$ have *M* entries, i.e. one entry per node. We store the coordinates in matrix $\mathbf{N}$ such that $N_{ij}$ is the $i\hbox {th}$ entry of the $j\hbox {th}$ eigenvector, corresponding to the $j\hbox {th}$ coordinate of node $n_i$.

In typical use cases from applications the matching problem is effectively two dimensional such that the first two eigenvectors of the Laplacian already capture sufficient information. Consider for example a factory floor where the variation is much larger in the two horizontal dimensions than in the vertical. However, it is required to take the third eigenvector into account in problems where the third dimension is relevant.

In some other cases, taking other eigenvectors into account may also be necessary. The diffusion map is essentially a harmonic analysis of networks and its use in localization relies on using eigenvectors that span the main dimensions of physical space. The first eigenvector always spans the longest dimension of the system. However, if the positions are in a layout that has significantly different length scales (for instance, if the positions are on the boundary of a narrow rectangle), the eigenvector that encodes information about the location in the secondary direction may not be the second eigenvector as harmonics of the major direction might have smaller eigenvalues. In such cases taking a small number of additional eigenvectors beyond the second, including the eigenvector for the shorter dimension, into account solves the problem. Our results (below) indicate that this is a very minor issue in practice as even in very long and thin geometries accurate matching is possible without the eigenvector for the short dimension unless the nodes form a perfect lattice. In the examples below we use the two eigenvectors of $\mathbf{L}$ corresponding to the two dimensions of the layout such that $\mathbf{N}$ is an $M\times 2$ matrix, unless noted otherwise.

We repeat the above process for the predefined positions. The distance matrix is now formed using the distances between the known positions

7 $$\begin{aligned} D'_{ij}= \left\Vert p_i-p_j\right\Vert . \end{aligned}$$

We construct the corresponding similarity matrix and normalized Laplacian as above and use the same Laplacian eigenvectors as our new coordinates for the positions. We denote the $j\hbox {th}$ coordinate of position $p_i$ as $P_{ij}$ which is analogous to $N_{ij}$ for the nodes.

Using the diffusion map we have found the new coordinates of the nodes $\mathbf{N}$ and positions $\mathbf{P}$. The matching can now be done in terms of these new coordinates. Because the identification of the new coordinates is based on eigenvector computation, information about the sign of the axes is lost. Hence, coordinates for nodes can have opposite signs (but are not scaled) with respect to the corresponding coordinates for positions. While this slightly complicates matters, it is a very small concern for the actual application. If we know the position of one of the nodes, we can compare the signs of the node’s coordinates with those of the corresponding position. If any of the coordinates differs in sign, we invert the signs of the respective coordinate entries for all nodes (i.e. the entries in the respective column of $\mathbf{N}$).

Even in the case where we don’t have one known node we can run the matching multiple times for the different axes coordinate signs and compare the quality of matching. If we are working in two dimensions that means the matching needs to be done 4 times (axis 1 inverted, axis 2 inverted, both inverted, none inverted) and the orientation that leads to the best match (see below) is picked as the result. In practice, this solves the problem unless the configuration of positions is fundamentally ambiguous (e.g. forming a symmetric lattice), in which case the correct matching can be picked from the, typically 4, alternatives by testing the assignments in one node.

For the matching we compute the Euclidean distances $E_{ij}$ between the locations of node $n_i$ and position $p_j$ in terms of the diffusion coordinates. We then organize nodes and positions into assignment pairs such that the sum of the distances within pairs is minimized. For this purpose we use the Kuhn–Munkres algorithm, commonly referred to as the Hungarian algorithm^28,29^.

The Hungarian algorithm is used to find a minimum weight matching in the bipartite graph where nodes and positions are vertices and $E_{ij}$ is the weight of the edge that can be placed between $n_i$ and $p_j$. The algorithm then uses dynamic rebalancing of the edge weights to identify a set of links of minimal weight that connects each node to exactly one position.

For the method proposed here, the Hungarian algorithm is the rate-limiting step that determines computational complexity. The algorithm can be implemented with a time complexity of ${\mathcal {O}}(M^3)$. For very large problems it might be advantageous to switch to alternatives to the algorithm such as one proposed by Ramshaw and Tarjan^30^ with a running time of ${\mathcal {O}}(M^2\sqrt{M}log{}(M))$. However, we expect that the efficiency of the method, as proposed here, will be sufficient for most applications. A reasonably efficient implementation on a standard desktop computer should be able to quickly match many thousands of nodes.

We use MATLAB to carry out the simulations. We start by defining a position layout and its associated distance matrix. Next, we randomly assign sensor nodes to positions. We calculate noiseless RSSI values from the distance matrix between the nodes. We then add Gaussian noise to RSSI values and calculate the noisy distance matrix $\mathbf{D}$. The signal-to-noise ratio (SNR) is calculated as signal mean over the standard deviation of the noise. Localization accuracy is evaluated as proportion of nodes assigned to correct positions. We repeat each experiment corresponding to each SNR value 100 times and report the mean accuracy and the 99% confidence interval.

## Results

**Figure 1:**
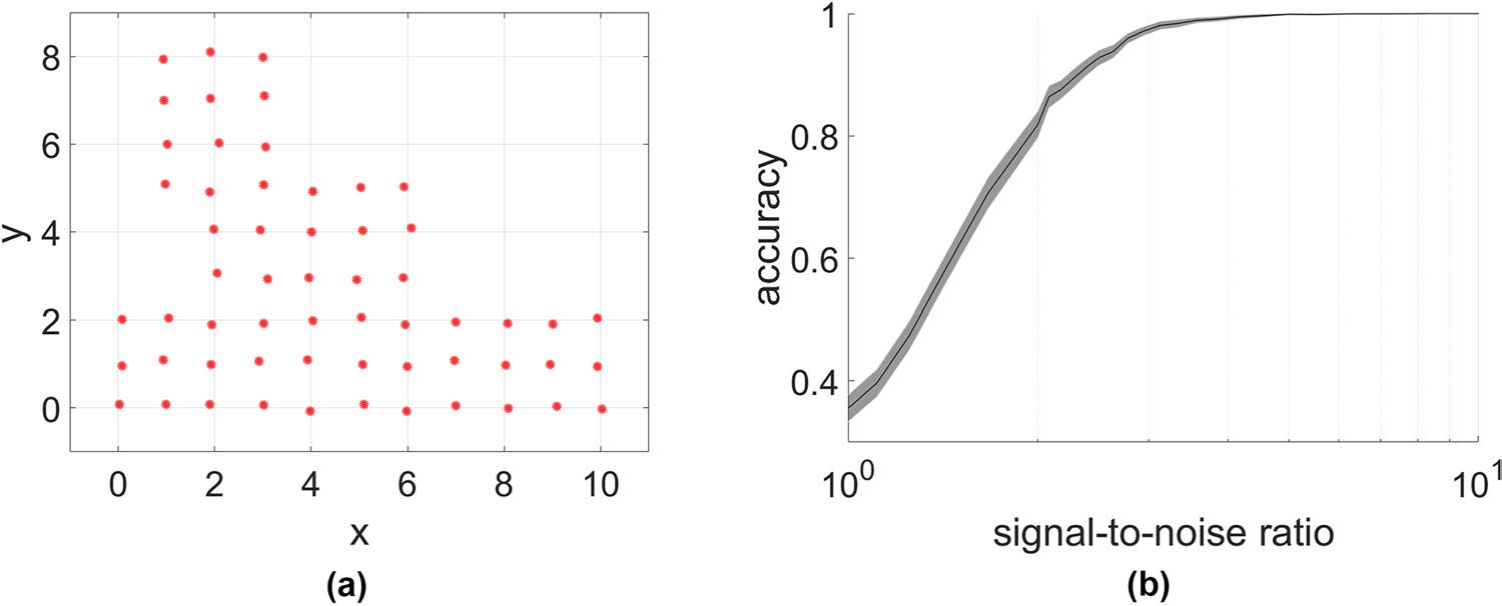
Performance in a realistic scenario. Shown are node positions (**a**) and the achieved accuracy in terms of signal-to-noise ratio (**b**). The curve is a mean over 100 realizations of the noise. The area around the curve represents a 99% confidence interval. In such realistic scenarios the method achieves perfect or near perfect matching results even at low signal-to-noise ratios of around 5.

As a first test we consider a system with 58 positions arranged in a plausible layout for a factory floor (Fig. 1a) with the inverse square law propagation model. As expected, using one node to align the eigenvectors reduced the computation time but lead to exactly the same results as picking the best of the four candidate assignments. The algorithm yields good accuracy even at moderate SNRs (Fig. 1b). At an SNR greater than 5, it is almost certain that the correct matching is retrieved.

**Figure 2:**
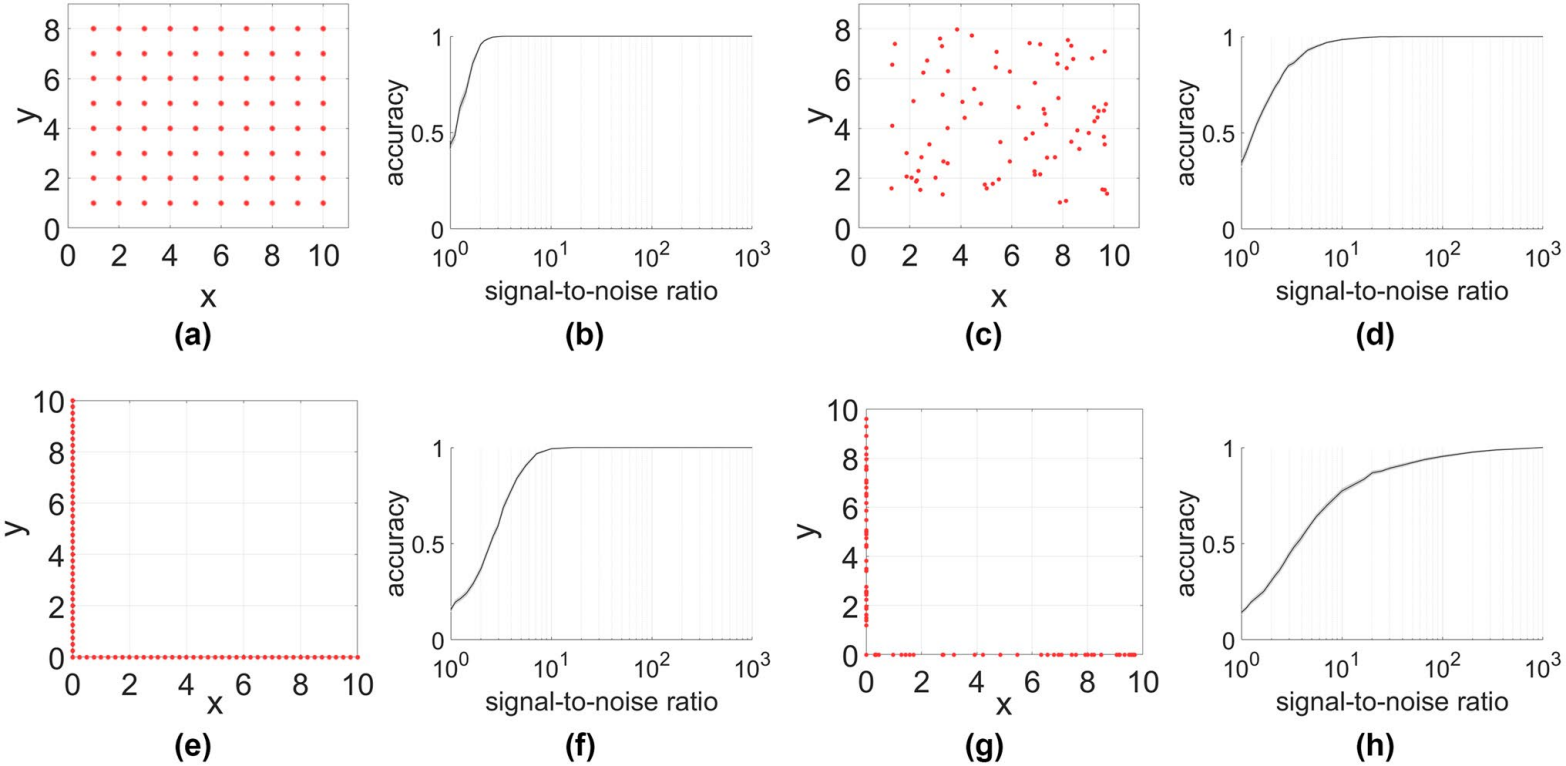
Performance in different artificial scenarios. Shown are node positions and matching accuracy analogously to Fig. 1. Matching is harder in biaxial layouts (**e**–**h**) than in uniform layouts. Highly random layouts (**c**,**d**,**g**,**h**) introduce an additional difficulty because they contain some positions that are very close together. Even in these intentionally difficult scenarios good matching results can be achieved if the signal-to-noise ratio is sufficiently high.

To understand possible sources of failures we now investigate some intentionally difficult cases. We consider 4 different layouts where 80 positions are placed in a 2-dimensional grid (grid layout) and randomly in a 2-dimensional plane (random 2D layout), and 81 positions are placed equidistantly along both coordinate axes (uniform biaxial layout) and randomly along the coordinate axes (random biaxial layout). Figure 2 shows the layouts and accuracy results for each case. The two random layouts are harder to match because they contain some positions that are very close together and hence easy to confuse. Proximity of nodes also explains why accuracy is less in the biaxial layouts. However, in all of these cases the method still achieves perfect or near-perfect results at moderate SNRs.

**Figure 3:**
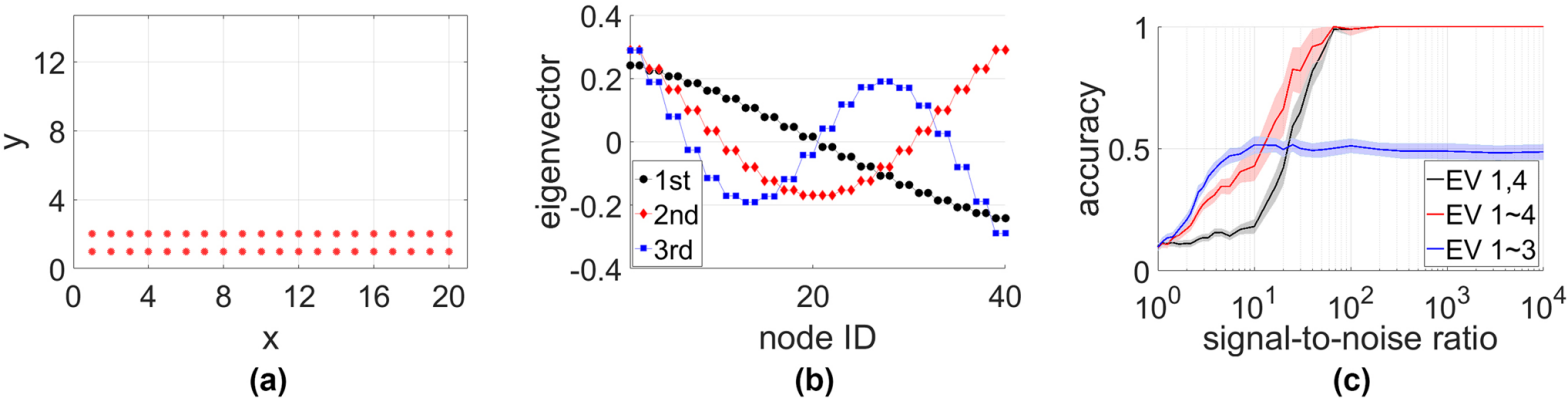
Usage of additional eigenvectors. If positions form long and thin lattices (**a**) information about the location along the shorter lattice direction is only contained in higher eigenvectors (**b**), as the first three eigenvectors assign almost the same value to pairs of points. Hence the accuracy plateaus at around 50% if only the first three eigenvectors (blue) are used for matching (**c**). However, using the first and the fourth eigenvectors (black) or all of the first 4 eigenvectors (red) yields good results.

In the “Methods” section we mentioned that complications can arise from the presence of harmonic eigenvectors. To illustrate these we study a system of 40 positions in a strip layout (Fig. 3a). In this case the first eigenvector contains information about the position of nodes along the primary axis of the strip. Hence this eigenvector cannot be used to differentiate between nodes that only differ in the secondary axis position (Fig. 3b).
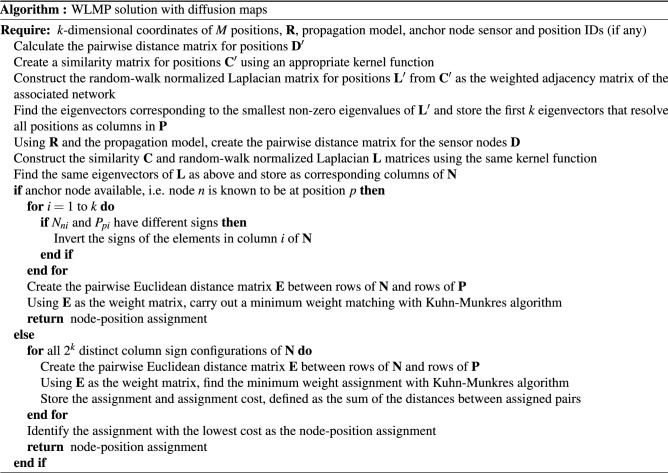

The second and third eigenvectors are higher harmonics of this eigenvector and thus contain essentially the same information. While these eigenvectors can be taken into account for more accurately locating a node in the primary direction, even using all 3 eigenvectors does not let us distinguish between nodes that lie side-by-side in the strip. Hence the matching accuracy plateaus at around 50% if these eigenvectors are used in the matching (Fig. 3c).

The first eigenvector that contains information about the position along the secondary axis is eigenvector 4. Hence using either the first and fourth eigenvectors, or all of the first 4 eigenvectors yields good matching results.

**Figure 4:**
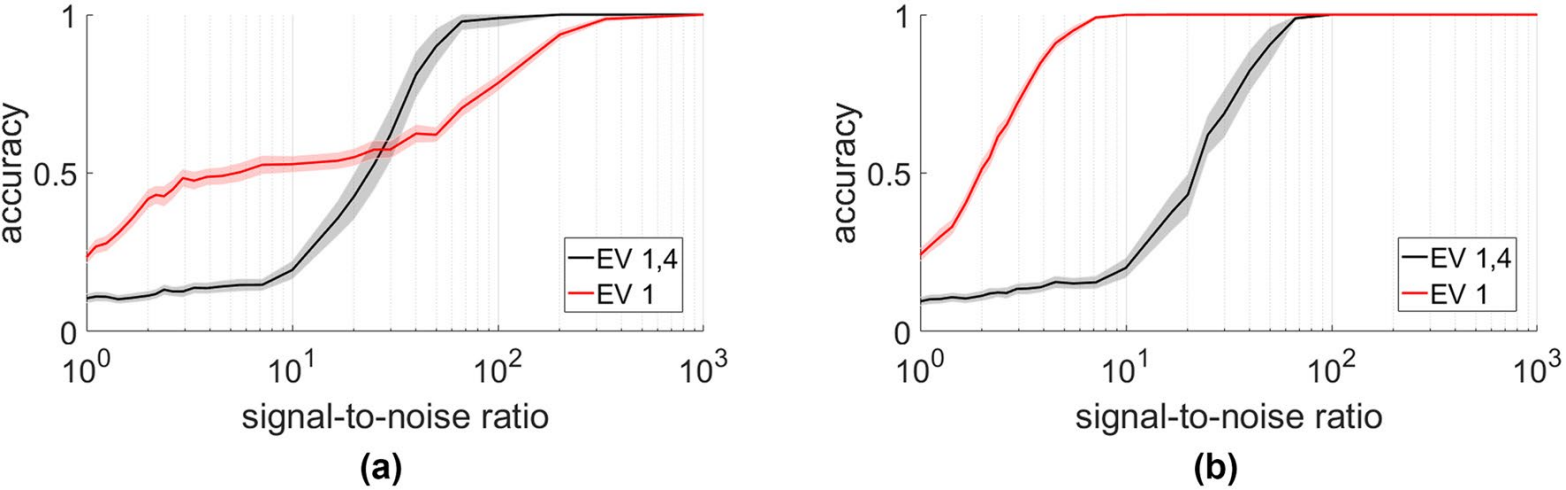
Small shifts in a lattice mitigate the effect of harmonics. We consider the layout from Fig. 3a, but shift one of the rows of positions by either 0.01 units (**a**) or 0.5 units (**b**). While this has little effect on the matching using eigenvectors 1 and 4 (black) already a small shift significantly improves the matching when only eigenvector 1 is used (red). At small signal-to-noise ratios or for larger shifts, using the first eigenvector yields better results.

We note that such problems due to harmonics only arise in the relatively artificial situation where the two rows of the strip are perfectly side-by-side. As we shift one of the rows even by small amounts, using only the first eigenvector yields an increasingly accurate matching, possibly even surpassing the accuracy for when considering the first and the fourth eigenvectors (Fig. 4).

We expect that the problems caused by the presence of harmonics will only play a minor role in applications. However, the possibility that such problems might occur highlights the need to select appropriate eigenvectors for the matching. The choice of eigenvectors to use can be made by checking whether a given set of eigenvectors can resolve all positions. Thus the backhaul server could determine a suitable set of eigenvectors once the blueprint is made available.

**Figure 5:**
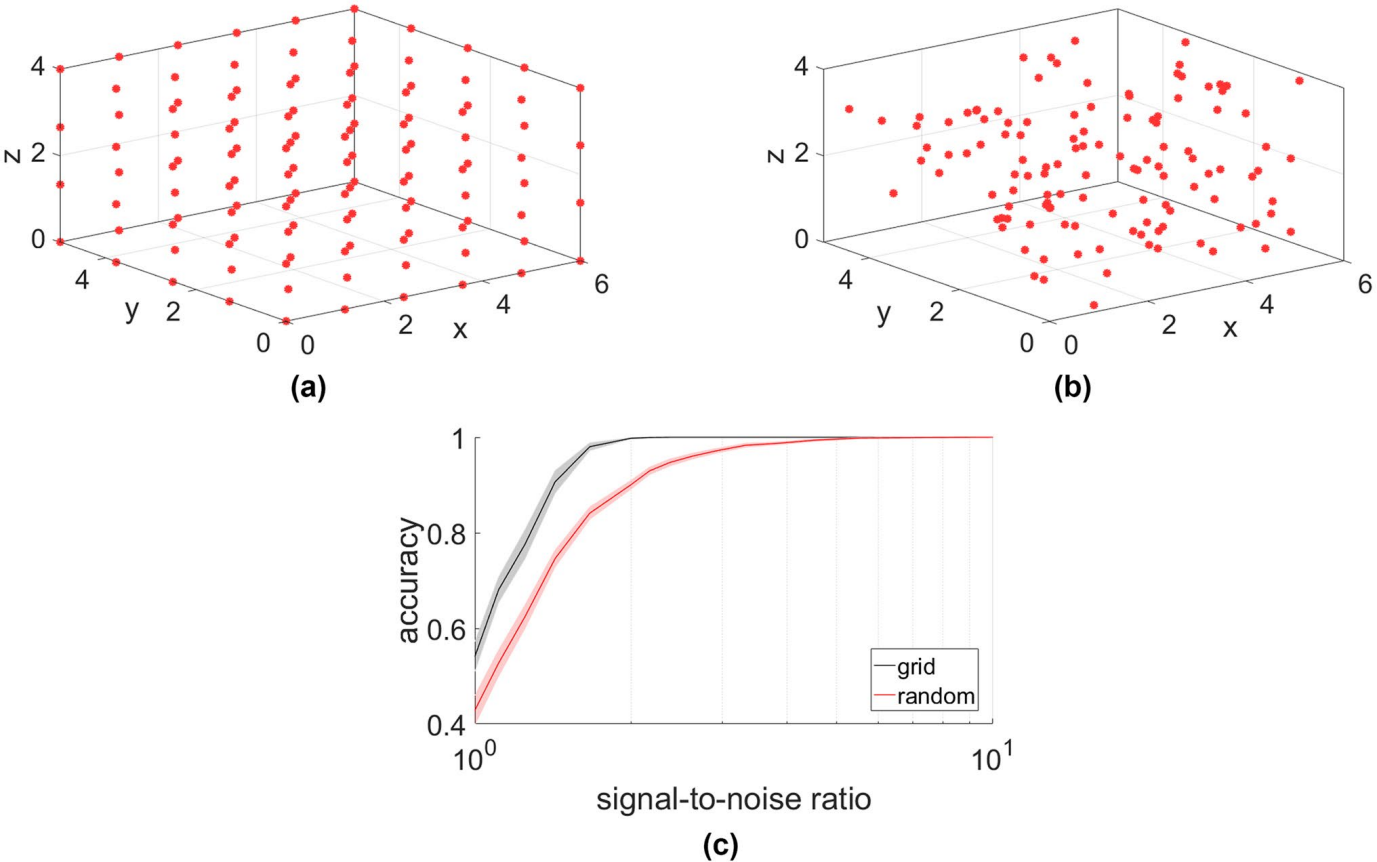
Localization performance in 3D problems. Node positions for grid (**a**) and random (**b**) layouts are shown. The accuracy plots (**c**) illustrate that the method can be implemented in solving 3D problems.

Next we investigate problems in 3D. We consider 120 positions that are either placed randomly or form a 3-dimensional grid. Location matching, using the first 3 eigenvectors, yields in both cases accurate results even at low SNR (Fig. 5). The accuracy thus surpasses that of the 2D layouts. This increase in accuracy is explained because the network of measurements is denser in 3D (i.e. lower effective diameter of the node graph) and a lesser chance that nodes are placed very closely together in the random layout.

**Figure 6:**
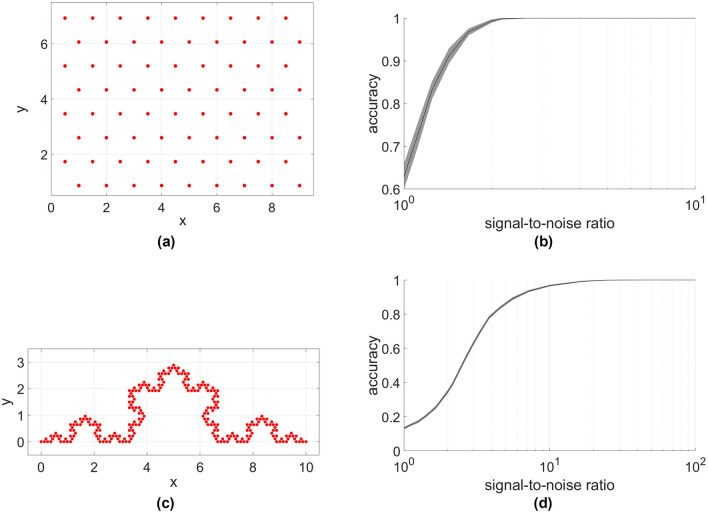
Performance using the log-distance path loss model. A hexagonal lattice (**a**,**b**) and a Koch curve with 4 iterations (**c**,**d**) layouts were considered. The method results in accurate matching in both layouts. The more varied length scales in the fractal compared to the lattice configuration results in less accuracy in smaller signal-to-noise ratios.

In our final simulations we use the log-distance path loss model with $\eta =3$ and $a=-50$ as a typical value for wireless sensors. We take a 72 node hexagonal lattice (Fig. 6a) as well as a 257 node Koch curve with 4 iterations (Figure 6c). The method yields an accurate matching for both of these layouts, with matching accuracy for the lattice (Fig. 6b) surpassing that of the Koch curve (Fig. 6d) due to the larger range of length scales in the fractal arrangement.

## Discussion

In this paper we proposed a solution to the Wireless Localization Matching Problem. The method is easy to interpret. In essence, it consists of mapping the position coordinates and noisy sensor node coordinates to a new space spanned by the principal directions of the data manifold implied by the pairwise distances (1), taking the projection of the data onto the first few coordinates as an optimal approximation of the new coordinates (2), and carrying out the bipartite matching in the new space where explicit coordinate data are now available for both sensor nodes and positions (3).

The key innovation was devising the first two steps, where we used diffusion maps to embed the original data points in the new space with the desirable coordinates. Due to the robustness of the mapping to noise as well as its optimality in terms of faithfulness to the original pairwise relationships, the method results in perfect or very accurate matching in numerous scenarios. We demonstrated in numerical simulations that the proposed algorithm can solve the matching problem without errors in realistic examples if the signal-to-noise ratio exceeds a threshold that is typically between $10^1$ and $10^2$. We believe this is sufficient to allow robust matching in applications.

Compared to several alternative methods in addressing the WLMP, the present approach is of lower computational complexity. A brute-force matching has a factorial time complexity and maximum likelihood matching methods involve a polynomial time likelihood calculation for each hypothesis corresponding to an assignment as well as an optimization algorithm like mixed integer programming or genetic algorithm whose complexity might depend on initial parameters and will at worst be of exponential time^4^. The proposed approach, however, has a third-order polynomial time complexity when implemented with the Hungarian method.

We emphasize that our algorithm does not require information beyond the blueprint of potential positions of equipment and pairwise signal strength measurements between proximal wireless sensors. In contrast to previous approaches^17^ for related problems, the method proposed here does not require anchor nodes, except in the case of fundamentally ambiguous layouts, where one node needs to determine the orientation of eigenvectors. It thus uses only information that would typically be accessible in the envisioned applications. Likewise, the numerical demand is such that even for very large systems it can be met with readily available desktop hardware.

When applying the algorithm, care has to be taken to take the right number of eigenvectors into account. However, it is easy to work out the right number by mapping the positions and testing for modal relationships among the first few eigenvectors. While it is thus essential that applications of the method should include a preprocessing step in which suitable eigenvectors are picked, this step could be straightforwardly integrated in software.

The examples considered in our tests focused on intentionally difficult cases. Configurations of positions in the real world are likely to result in more accurate assignments than some of the extreme scenarios considered here. We expect that similar to the example in Fig. 1 perfect matching can already be achieved at a signal-to-noise ratio of about 5. We also expect that the main ideas in the method can be extended to include more general cases where bipartite matching and localization tasks are involved.

We hope that the proposed method will help to realize the future applications of wireless localization. Besides numerical simulations, future physical experiments will potentially yield further insight for assessing the feasibility of the method as well as its implementation details. We anticipate that future refinements of this approach will lead to additional improvements. Such refinements may include the use of improved models in the construction of the distance matrix or the use of a thresholding step, which is commonly used in other applications of the diffusion map. However, the fine-tuning of the additional parameters introduced by these refinements as well as the benefit conveyed by them will likely depend on the specific application.
